# Short-Term Effects of Specific Sensorimotor Training on Postural Assessment in Healthy Individuals: A Pilot Study with a Randomized Placebo-Controlled Trial

**DOI:** 10.3390/jfmk8020046

**Published:** 2023-04-19

**Authors:** Donatella Di Corrado, Vincenzo Cristian Francavilla, Rosamaria La Paglia, Maria Chiara Parisi, Andrea Buscemi, Marinella Coco

**Affiliations:** 1 Department of Sport Sciences, Kore University, Cittadella Universitaria, 94100 Enna, Italy; 2 Department of Medicine and Surgery, Kore University, 94100 Enna, Italy; vincenzo.francavilla@unikore.it (V.C.F.); mariachiara.parisi@unikore.it (M.C.P.); 3 Department of Psychology, Kore University, 94100 Enna, Italy; rosamarialapaglia@gmail.com; 4 Study Center of Italian Osteopathy and Horus Social Cooperative, 95100 Catania, Italy; andreabuscemi@virgilio.it; 5 Department of Education Sciences, University of Catania, 95124 Catania, Italy; marinella.coco@unict.it

**Keywords:** posture, sensorimotor system, stability, short-term

## Abstract

It is well-known that sensorimotor training aims to increase the performance of the sensorimotor system to maintain an upright position. Through the use of a randomized placebo-controlled trial, the specific aim of this study was to investigate the short-term effects of a specific session of sensorimotor training on postural balance, stability and coordination in healthy, recreationally active participants. Ninety subjects were randomly allocated into three groups: experimental (*n* = 30), placebo (*n* = 32) and control (*n* = 28). The experimental group performed a 5 min warm-up, with the sensorimotor training consisting of 60-min specific sensorimotor exercises; the control group was not allowed to perform any sensorimotor training; the placebo group observed a video clip of an individual belonging to the experimental group performing the sensorimotor training accurately. All participants were seen three times per week for 4 weeks. Before and after the entire training, all groups of participants undertook stabilometric parameter assessment. The intervention-mediated sensorimotor training confirmed significant enhancement in the proprioceptive system. Significant improvement in the motor and/or sensory function was observed in the experimental and placebo groups. In conclusion, our findings suggest that specific sensorimotor training performed 3 days per week for 4 weeks could improve postural balance, stability and coordination in healthy individuals.

## 1. Introduction

Balance is generally defined as the ability to maintain an upright position in a precise spatial orientation or to recuperate equilibrium after external dynamic perturbations, and it is in relation with the inertial forces acting on the body and the inertial features of its segments. Additionally, balance is a crucial component of normal daily activities (e.g., walking, running and climbing stairs), and in the prevention of falls and resulting injury [1]. Even a small movement of a part of the body is an element of a complex pattern of muscular activity, which includes both muscles directly producing the observed movement and muscles remotely located from the moving part. Therefore, to successfully perform a variety of motor skills, we must able to coordinate movements of different body parts. Coordination allows an individual to use the neuromuscular and kinesthetic senses of body parts to perform movement successfully and with gracefulness [2]. Preserving postural balance under static or dynamic conditions is indispensable for quotidian life activities [3].

The ability to control balance is based on the combination of sensory information from the somatosensory, visual and vestibular systems, which work together with the neuromuscular system to control body configuration and to stabilize the body’s center of mass, even in the presence of forces that would usually change the condition [4]. Balance takes two different forms: static and dynamic. Static balance is considered to be the ability to maintain an upright posture and to preserve the line of gravity within the limits of the base of support. Dynamic balance is considered to be the ability to maintain stability during weight shifting, often while changing the base of support [5]. Maintaining body equilibrium depends on proprioceptive information resulting from three areas: the sole of the foot, the cervical spine and the sacroiliac joint. Input from the sensorimotor system is then combined with information from the eyes and inner ear to control postural balance [6]. For instance, the usual aptitude to close one’s eyes while standing without loss of postural equilibrium comes from the ability of the somatosensory and vestibular perceptions to contribute adequate afferent information despite the lack of visual information [7].

It is well-known that sensorimotor training aims to increase the performance of the sensorimotor system. It can be characterized as a progressive balance training program using labile surfaces to elicit automatic postural stabilization. Exercises and devices for balance training should not be implemented casually or haphazardly [8]. Each exercise should elicit automatic and reflexive muscular stabilization, challenging the subject to maintain postural control under a variety of situations. Previous studies have suggested that different types of generalized training may be directly related to improvement of visual skills [9], spatial orientation sensitivity [10], the rate of force development [11] and motion sensitivity [12].

Several interventions have been developed for the preventing different sports injuries, aiding in rehabilitation and improving postural abilities, proprioception, and performance [13,14,15,16].

Sensorimotor training is primarily incorporated into rehabilitation programs in athletic populations and in fall prevention programs for the elderly [17,18,19]. Thus, several studies have explored his effects mainly on athletes who want to improve performance and prevent injuries and on clinical populations [20,21]. The skill to control and maintain balance is often taken for granted; essentially, it provides the primary foundation for mobility, for the use of the upper extremities and for maintaining overall functional independence throughout life. For all ages, the absence of apposite control of balance and posture can have negative effects on both mental and physical health, such as a lowered ability to perform physical tasks, loss of independence and interruption in social activities [1]. The principal goal of sensorimotor training is to increase muscle reaction and restore the automatic reflexive stabilization for dynamic restraint, rather than to increase total joint strength. In this regard, it clearly has significant role in improving postural balance and stability; however, it is still unclear whether integrating specific sensorimotor tasks into a training program increases postural balance, stability and coordination in a more general way in a healthy population [22,23].

Using a randomized placebo-controlled trial, we aimed to investigate the effects of a specific session of sensorimotor training on postural balance, stability and coordination in healthy, recreationally active participants. This intervention, which was developed by the physical therapist Pietro Tamburo (https://metodotamburo.com/ (accessed on 28 March 2023)), aims to promote proper integration of the proprioceptive and exteroceptive afferents in all information channels and to thus eliminate neuromuscular imbalances. Subsequently, it aims to provide a correct mental organization for walking activity to create a new sense of awareness of one’s body and regain optimization not only of posture, but also of mental and physical well-being. We hypothesized that the specific dosage of interventions of this sensorimotor training would show potential effects on outcomes of postural balance, stability and coordination. We further hypothesized that the experimental group would show significant improvement of these variables as compared to the control and placebo groups.

## 2. Materials and Methods

### 2.1. Sample Size Calculation and Participants

**Figure 1:**
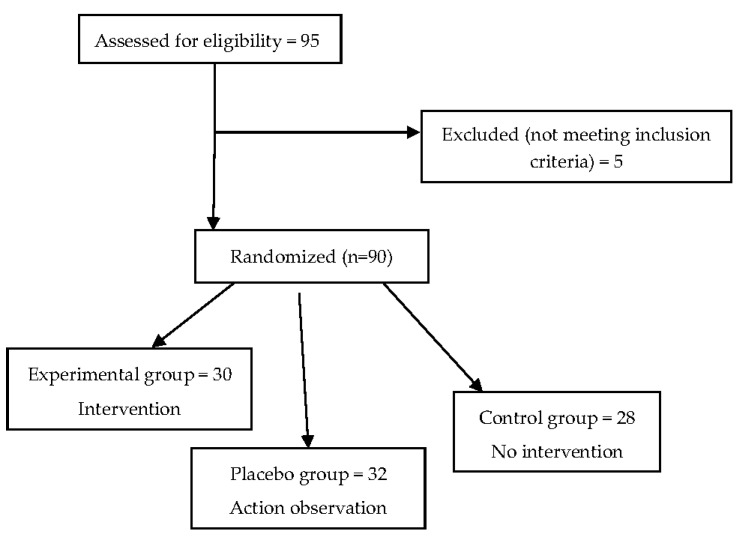
Flowchart of the study.

This study was a randomized placebo-controlled trial. Group sample sizes were decided by power analysis, with G*Power being used in a one-way between-subjects ANOVA [24]. The input parameters were effect size (ŋ^2^ = 0.70), alpha level of 0.05, power (0.95%) and the number of groups sampled (three groups). Finally, the result revealed the need for 25 participants in each of the three groups (75 in all). Therefore, a total of n = 95 participants were recruited to take part in the research (Figure 1). The inclusion criteria were as follows: absence of motor or neurological deficits, no trauma, no pain and no orthopedic injury or vestibular impairment. The exclusion criteria were those currently participating in a structured balance exercise program and those involved in professional sports. Five participants receiving physical therapy were not tested. Ninety healthy, recreationally active people, aged between 21 and 27 years (M = 23.7, SD = 1.75), were randomly assigned to one of the three groups: experimental (n = 30), placebo (n = 32) and control (n = 28).

All participants were new to this type of training, had normal or corrected-to-normal vision and performed recreational physical activity between 2 and 3 times per week. The study population was recruited after the lectures from the University of Physical Education. All participants were provided with a full explanation of the protocols prior to commencement of the study and then signed an informed consent form for all tests. This study was carried out in accordance with the recommendations of the Ethical Code of the University of Palermo and of the Code of Ethics approved by the General Assembly of the Italian Association of Psychology held on 27 March 2015. All the procedures were conducted in accordance with the ethics of the Declaration of Helsinki, and the University Enna Kore Internal Review Board for psychological research (UKE-IRBPSY-03.22.13) provided ethical permission.

### 2.2. Procedures

All participants were submitted to a postural assessment. Measurements were conducted before T0 (baseline) and after the T1 the intervention period and were performed in the same laboratory with analogous conditions (room temperature 21 °C, electric illumination and time of day) but by a different investigator who was blinded to the interventions received by the participants. All participants were seen three times per week for 4 weeks. Days were different for all three groups to avoid them finding out which group they belonged to.

#### 2.2.1. Intervention

##### Experimental Procedure

The experimental group (*n* = 30) performed the sensorimotor training developed by the physical therapist Pietro Tamburo under the supervision of the same investigator at same time of the day (between 9.00 and 13.00). The whole training was performed for 4 weeks (three sessions per week) in a total of 12 sessions, and each training session consisted of 60 min sessions of specific sensorimotor exercises, including a 5 min warm-up and a 5 min cool-down. In each session, a 5 min rest was given between different types of exercises. The objective of this protocol was to stimulate both static and dynamic balance, with the overall strengthening of the muscles and the stabilization of the ankle and foot joints. Some of the exercises consisted of changing arm position, opening/closing eyes or moving in a different direction (see Table 1).

##### Placebo

Several studies have shown that the action observation (i.e., watching movements performed by others) can stimulate the corresponding motor representation and can induce the similar alterations in skills as seen in physical exercise [25,26,27]. For example, the brain can realize an interior simulation of the specific motor training for the observed actions. Accordingly, the placebo group (*n* = 32) observed a recorded video of an individual belonging to the experimental group performing the sensorimotor training accurately.

##### Control

The control group (*n* = 28) received a minimal intervention, consisting of various brochures with general information about the postural training and the observation of landscape pictures three times a week for four weeks.

### 2.3. Measurements

#### 2.3.1. Baropodometric Platform

Postural balance was measured using a footscan baropodometric platform (T-Plate, Molinari S.r.l., Piacenza, Italy) under the following parameters: platform’s surface 610 × 580 mm, with an active surface of 400 × 400 mm and 4 mm thickness. The participants were placed bipedally, with their bare feet side-by-side on the platform, each foot about 20 cm away from the other and without any type of other support. All subjects were oriented to maintain an upright and natural position, always looking at a fixed point in front of them for 51.2 s with open eyes. The software obtained the center of pressure (CoP) position in the medial–lateral direction (ML) and anterior–posterior direction (AP). It also measured the speed of movement of the CoP and the length covered by the CoP. Data were collected and analyzed using the software supplied with the platform, which continuously recorded the CoP trajectories at a sampling rate of 100 Hz.

#### 2.3.2. Platform I-moove

**Figure 2:**
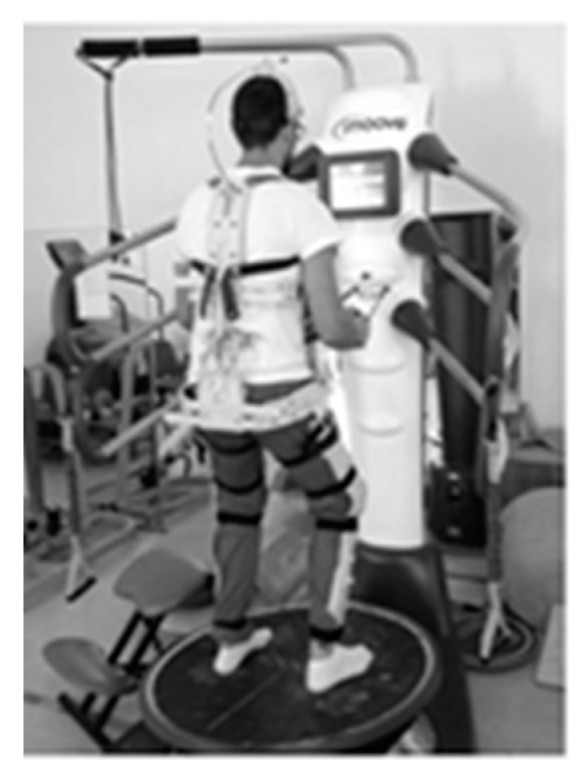
IMOOVE^®^.

The stability and coordination parameters were assessed using the IMOOVE^®^ system (Allcare Innovations, Chabeuil, France). Based on the exclusive Ellisferic movement (natural movement of the body of spiral rotation), it stimulates deep proprioception and vertebral natural movement to restore the muscular and postural balance of the body. To maintain balance, the participants must control the movements of the unstable platform, involving visual feedback in real time. Another feature of the Imoove system is to be able to assess the stability index and coordination index using the check-up program enabled by the presence of a monitor (Figure 2). To this end, Imoove was set to an intensity level of 2, a sensitivity level of 1 and a duration of evaluation of 60 s. The data used for the study included coordination index and stability index.

### 2.4. Statistical Analysis

Prior to the statistical analyses, assumptions on normality and on the homogeneity of variances were verified (Kolmogorov–Smirnov and Levene’s tests being nonsignificant in all cases; *p* > 0.05). This study was designed to show the improvement in postural assessment after the whole training period. One-way analysis of variance (ANOVA) was conducted to compare the differences (before and after the training) on the postural balance, stability and coordination parameters between groups. In cases of statistical significance, a Tukey’s test was also conducted for post hoc analysis. An effect size was used for each analysis with the eta-squared statistic (ŋ^2^) to evaluate the practical significance of findings. Effect sizes were presented using Cohen’s d, where d = 0.2 was considered to be a “small” effect size, d = 0.5 represented a “medium” effect size and d = 0.8 represented a “large” effect size [28]. All statistical analyses were processed with SPSS version 26 (SPSS Inc., Chicago, IL, USA) and are presented as mean ± SD (level of significance: *p* ≤ 0.05).

## 3. Results

### 3.1. Anthropometric Characteristics

The anthropometric characteristics of participants are summarized in Table 2.

### 3.2. Comparison of Postural Balance

Length, speed and center of pressure position in the medial–lateral direction (ML) and anterior–posterior direction (AP) are shown in Table 3.

Our data showed a significant improvement in the speed of center of pressure and in the length covered by center of pressure in the experimental and placebo group (respectively *p* = 0.02 and *p* = 0.001) as compared with the control group. Nevertheless, in both cases, there were no significant differences between the experimental and placebo groups (*p* > 0.05). In the mediolateral position and anterior–posterior position, the experimental group exhibited significant improvement compared with the placebo and control groups (*p* = 0.001), but there were no significant differences between the placebo and control groups (*p* > 0.05). Finally, the effect size was found to be stronger (*η^2^* = 0.36) in length covered by center of pressure.

### 3.3. Comparison of Stability and Coordination Parameters

**Figure 3:**
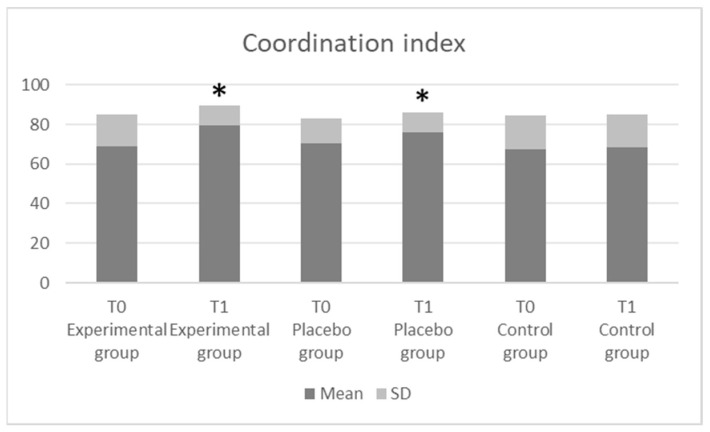
The coordination index results (IMOOVE^®^ system).

**Figure 4:**
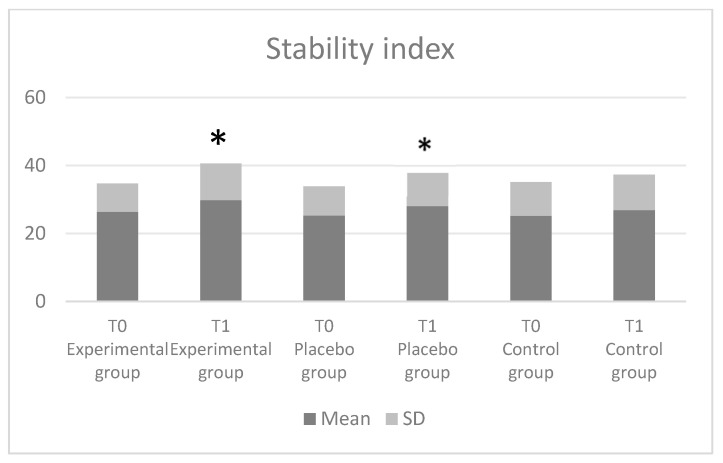
The stability index results (IMOOVE^®^ system).

The comparisons of the coordination and stability parameter indices are shown in Figure 3 and Figure 4, respectively. Our data revealed a significant improvement in the coordination index (*p* = 0.001; F = 73.19) and in the stability index (*p* = 0.001; F = 64.68) in the experimental and placebo groups as compared with the control group. Nevertheless, in both cases, there were no significant differences between the experimental and placebo groups (* *p* > 0.05). Finally, the effect size was found to be stronger (*η^2^* = 0.35) in the coordination index.

## 4. Discussion

The purpose of this study was to investigate the effects of a specific dosage sensorimotor training intervention on postural balance, stability and coordination in healthy, recreationally active participants. Our hypotheses were partially supported by obtained results. The study showed that this 4-week sensorimotor training intervention was associated with a significant improvement in static and dynamic balance from baseline in the experimental and placebo groups as compared to the control group. From an efficiency standpoint, in short- and long-term conditions, previous studies found an improvement in postural stabilization and balance parameters as a consequence of 4 weeks of specific sensorimotor training [29,30,31,32]. The experimental group doing the specific sensorimotor training showed significantly greater improvement in all the postural parameters assessed (postural balance, stability and coordination) as compared with the placebo and control group. First, according to our results, the length covered by center of pressure and the speed of movement of the center of pressure were significantly increased after the sensorimotor training in the experimental and placebo groups as compared to the control group.

According to Romero-Franco et al., specific sensorimotor training may allow the individual to gain better static and dynamic postural balance, preserving a dynamic integration of internal and external forces [33].

The motor activation in the placebo group may result from sensorimotor learning. According to Catmur et al. [34], sensorimotor training alters observation-related motor activation, showing that the observation of movements is enough to execute new motor kinematics. Our findings suggest the ability to observe and understand someone else’s action lets us to recognize what the observed agent is doing. It is therefore likely that action observation leads to organizational changes in the brain which may be directly associated with motor learning [35]. Behrendt and colleagues [36] supported the neurophysiological basis of these results. In fact, they demonstrated that healthy individuals observing another person walking reproduce a mental representation of the observed gait which they recreate concomitantly in lower-limb EMG recordings. Observing the sway of others can also influence current and future postural tasks. In research conducted by Taube and colleagues [37], subjects watched videos of an actor performing balance exercises for four weeks. At the end, observers performed a balance task on a free-moving platform and used the same techniques they had observed, significantly reducing their postural sway in the task.

Moreover, a statistically significant improvement of mean mediolateral position (ML) and mean anterior–posterior position (AP) was verified only in the experimental group as compared to placebo and control group. This position-specific benefit might be strongly associated with the movement of training exercises which were utilized in this study. In line with our results, Strang et al., found improvements on postural sway in both the mediolateral and the anterior–posterior positions after specific sensorimotor training [38]. Under conditions of upright stance, with eyes open, research has shown that somatosensory input from the legs likely brings the most accurate sensory feedback used for postural balance, followed by feedback from vision and lastly, the vestibular system [39].

Finally, the coordination index and stability index were significantly increased after the sensorimotor training in the experimental and placebo groups as compared to the control group. Specifically, the sampled individuals with intervention improved the ability to maintain an optimal posture on the Imoove system with a balancing platform with hemispheric movement, which requires continuous realignment of the surfaces and body. These results are in line with previous reports documenting an improvement of static balance after a specific training program [23,40].

No significant improvement in motor and/or sensory function was observed in the control group.

There are some limitations to this study: (a) only the short-term effects of the sensorimotor training were observed; (b) no influence on the repeatability of the objective evaluation of tests used is shown. To strengthen this hypothesis, further studies should assess the therapeutic effect on posture and balance of this specific sensorimotor training program, both in the short- and long-term, using the same evaluation methods that we used in this study. Once it has been demonstrated that this kind of sensorimotor adaptation training is successful in providing improved postural balance, stability and coordination, the information gained from future studies could then be used to develop an effective fall and injury prevention program.

## 5. Conclusions

Balance is an indispensable component for overall physical performance. Greater stability and balance may benefit the activity of daily living by providing a foundation for greater force production in the upper and lower extremities. The current study demonstrated that the *TAMBURO* method-mediated sensorimotor training provided significant enhancement in the proprioceptive system. Our findings suggested that the newly developed sensorimotor training could be a promising option to improve postural balance, stability and coordination and should be included in training routines. Satisfactory improvement was also observed in the placebo group. The action observation may have advantages over physical task performance when individuals are asked to reproduce multiple motor tasks. Nevertheless, observational learning is not as effective as physical motor learning.

## Figures and Tables

**Table 1:** Exercises of the sensorimotor training.

**Step 1: On the floor in the supine position**
**Ex. 1:** Barefoot walk, respecting the physiological progression of the step, toe walk (straight ahead, toes out, toes in) and forward backward swing (2 sets for each leg, each set 30 repetitions).
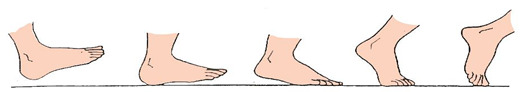
**Step 2: In the center of the proprioceptive mat**
**Ex. 2:** The subject should stand, with arms along the hips and feet together, and move the head forward and backward (5 times with eyes open and 5 times with eyes closed). The subject should move the head laterally to the right and to the left (5 times with eyes open and 5 times with eyes closed).
**Ex. 3:** The subject, with arms at his or her sides, must maintain balance on one foot and lift the other leg forward bending the knee to 90° and then repeat the exercise by bringing the leg back by bending the knee to 90° and finally repeat the exercise with the contralateral foot (5 s with eyes open and 5 s with eyes closed).
**Ex. 4:** The subject, resting on one foot only, should swing the other leg forward and backward and repeat the exercise while resting on the contralateral foot (15 s with eyes open and 5 s with eyes closed).
**Ex. 5:** The subject, resting on one foot only, should extend the other leg forward to first internally and then externally rotate the ankle, and repeat the exercise resting on the contralateral foot (10 s eyes open for each movement and 5 s eyes closed for each movement).
**Ex. 6:** The subject, resting on one foot only, should flex the knee at 90° and, from this position, first internally and then externally rotate the ankle and repeat the exercise resting on the contralateral foot (10 s with eyes open for each movement and 5 s with eyes closed for each movement).
**Ex. 7:** The subject, resting on one foot only, must first internally and then externally rotate the hip of the other leg and repeat the exercise with the contralateral limb (10 s with eyes open and 5 s with eyes closed).
**Ex. 8:** The subject, still in the supine position, should lift the right leg and arm left at the same time and repeat the exercise with the leg and arm reversed (5 times with eyes open (3 sets) and 5 times with eyes closed (3 sets)).
**Step 3: On the floor in the supine position**
**Ex. 9:** The subject, resting on both feet, must stand up on his or her toes and first with the arms along the sides and then with the arms raised upward (5 s with eyes open and 5 s with eyes closed).
**Ex. 10:** The subject, resting on both feet, should flex the torso forward until he or she can place both hands on the ground (if it is possible) and slowly return to the position initial. This should be repeated 5 times.

**Table 2:** Participants’ anthropometric characteristics. Mean (±standard deviations).

	Age [Years]	Height [m]	Weight [kg]	Gender
EG	23.9 ± 1.8	1.71 ± 4.86	81.7 ± 7.29	13 m, 17 f
PG	23.4 ± 1.7	1.78 ± 5.04	91.8 ± 6.32	18 m, 14 f
CG	23.8 ± 1.8	1.69 ± 4.36	77.4 ± 7.37	12 m, 16 f
*p*	0.871	0.693	0.782	0.614

Notes: EG is the experimental group, PG the placebo group and CG the control group. Statistically, no differences were found between these groups in terms of age, height or weight. The groups were equally well balanced in terms of gender.

**Table 3:** Comparison of postural balance parameters.

Test	Group	Pre-T0	Post-T1	Pre-Post	F	*η* ^2^	*Tukey*
Speed (m/s)	Experimental ^a^	78.25 ± 14.98	84.6 ± 21.46	−6.35 ± 3.48 ^†^	35.06	0.21	a = b > c
	Placebo ^b^	80.41 ± 18.51	83.51 ± 20.63	−3.1 ± 2.32 ^†^			
	Control ^c^	79.44 ± 19.46	80.96 ± 21.56	−1.52 ± 0.74			
Length (mm)	Experimental ^a^	324.79 ± 56.88	373.51 ± 97.52	−48.72 ± 23.48 *			
	Placebo ^b^	342.10 ± 76.56	361.19 ± 84.56	−19.09 ± 9.08 *	71.16	0.36	a = b > c
	Control ^c^	317.1 ± 53.57	321.9 ± 57.56	−4.08 ± 3.22			
ML (cm)	Experimental ^a^	4.22 ± 3.60	6.03 ± 2.24	−1.81 ± 0.48 *			
	Placebo ^b^	3.28 ± 2.21	3.78 ± 2.03	−0.5 ± 0.42	21.98	0.14	a > b = c
	Control ^c^	3.15 ± 2.29	3.59 ± 2.09	−0.44 ± 0.38			
AP (cm)	Experimental ^a^	28.12 ± 10.25	32.95 ± 11.75	−4.83 ± 2.11 *			
	Placebo ^b^	29.91 ± 11.04	30.6 ± 10.01	−0.69 ± 0.78	33.06	0.19	a > b = c
	Control ^c^	28.39 ± 11.56	29.09 ± 10.56	−0.7 ± 0.79			

Notes: Length is length covered by center of pressure; speed is speed of center of pressure, ML is mean mediolateral position, and AP is mean anterior–posterior position. ^†^ *p* < 0.05; * *p* < 0.001.

## Data Availability

The data that support the findings of this study are available from the corresponding author (D.D.C.) upon reasonable request.
